# Lack of gp130 expression in hepatocytes attenuates tumor progression in the DEN model

**DOI:** 10.1038/cddis.2014.590

**Published:** 2015-03-05

**Authors:** M Hatting, M Spannbauer, J Peng, M Al Masaoudi, G Sellge, Y A Nevzorova, N Gassler, C Liedtke, F J Cubero, C Trautwein

**Affiliations:** 1 Department of Internal Medicine III, University Hospital, RWTH Aachen, Aachen, Germany; 2 Institute of Pathology, University Hospital, RWTH Aachen, Aachen, Germany

## Abstract

Chronic liver inflammation is a crucial event in the development and growth of hepatocellular carcinoma (HCC). Compelling evidence has shown that interleukin-6 (IL-6)/gp130-dependent signaling has a fundamental role in liver carcinogenesis. Thus, in the present study we aimed to investigate the role of gp130 in hepatocytes for the initiation and progression of HCC. Hepatocyte-specific gp130 knockout mice (gp130^Δhepa^) and control animals (gp130^f/f^) were treated with diethylnitrosamine (DEN). The role of gp130 for acute injury (0–144 h post treatment), tumor initiation (24 weeks) and progression (40 weeks) was analyzed. After acute DEN-induced liver injury we observed a reduction in the inflammatory response in gp130^Δhepa^ animals as reflected by decreased levels of IL-6 and oncostatin M. The loss of gp130 slightly attenuated the initiation of HCC 24 weeks after DEN treatment. In contrast, 40 weeks after DEN treatment, male and female gp130^Δhepa^ mice showed smaller tumors and reduced tumor burden, indicating a role for hepatocyte-specific gp130 expression during HCC progression. Oxidative stress and DNA damage were substantially and similarly increased by DEN in both gp130^f/f^ and gp130^Δhepa^ animals. However, gp130^Δhepa^ livers revealed aberrant STAT5 activation and decreased levels of transforming growth factor-*β* (TGF*β*), pSMAD2/3 and SMAD2, whereas phosphorylation of STAT3 at Tyr705 and Ser727 was absent. Our results indicate that gp130 deletion in hepatocytes reduces progression, but not HCC initiation in the DEN model. Gp130 deletion resulted in STAT3 inhibition but increased STAT5 activation and diminished TGF-dependent signaling. Hence, blocking gp130 in hepatocytes might be an interesting therapeutic target to inhibit the growth of HCC.

Hepatocellular carcinoma (HCC) represents a major health problem as it ranks fifth among the solid tumors and third among the worldwide cause of cancer mortality in males with half a million deaths per year.^1, 2^ The development of HCC is a multifactorial process that includes the accumulation of mutations in the cellular tumor-suppressive and tumor-promoting pathways as well as a disturbance in immune surveillance. It involves the transition of a normal cell to a preneoplastic lesion that develops into a malignant tumor. The driving pathophysiological mechanisms by which tumor initiation and progression occur during the process of hepatic carcinogenesis needs to be explored to target pathways, which are attractive for therapeutic intervention.

The progression of chronic liver disease and, at the end stage, the growth of HCC is associated with persistent tissue injury, leading to a chronic inflammatory response in the organ reflected by an imbalance in pro-inflammatory and anti-inflammatory cytokines.^3, 4, 5^ Members of the interleukin-6 (IL-6) family such as IL-6, oncostatin M (OSM), leukemia inhibitory factor (LIF) and cardiotrophin share the glycoprotein 130 (gp130) for signal transduction. Gp130 has been shown to have a fundamental role in the development, hematopoiesis, cell survival and growth as well as in infection, immunity and inflammation.^3, 6^ Especially, molecular studies of IL-6 dependent gp130 activation have significantly contributed to the understanding of the role of gp130 during infection and inflammation.^7^

Binding of IL-6 to the IL-6 receptor (IL-6R*α*) causes a heterodimeric association with gp130 to form a signaling complex composed of a gp130 homodimer and two IL-6R*α* molecules which, in turn, trigger the phosphorylation and activation of the JAK-STAT (signal transducer and activator of transcription) pathway initiating gene transcription.^7, 8, 9, 10^ We and others have previously demonstrated the relevance of gp130 in regulating cellular recruitment to local sites of inflammation.^7, 11, 12, 13^ In addition, there is good evidence that gp130 might have a role in cancer, as elevated IL-6 levels are associated with higher risk of developing certain tumors.^14^

In the present study, we aimed to investigate the role of gp130 in hepatocytes for the initiation and progression of HCC during inflammation-triggered carcinogenesis. We thus used the diethylnitrosamine (DEN)-induced HCC model which recapitulates human HCC with poor prognosis^14, 15^ and addressed the role of gp130 in DEN-induced tumorigenesis by using animals with hepatocyte-specific gp130 deletion.

## Results

### Acute inflammation is ameliorated in gp130^Δhepa^ mice

**Figure 1:**
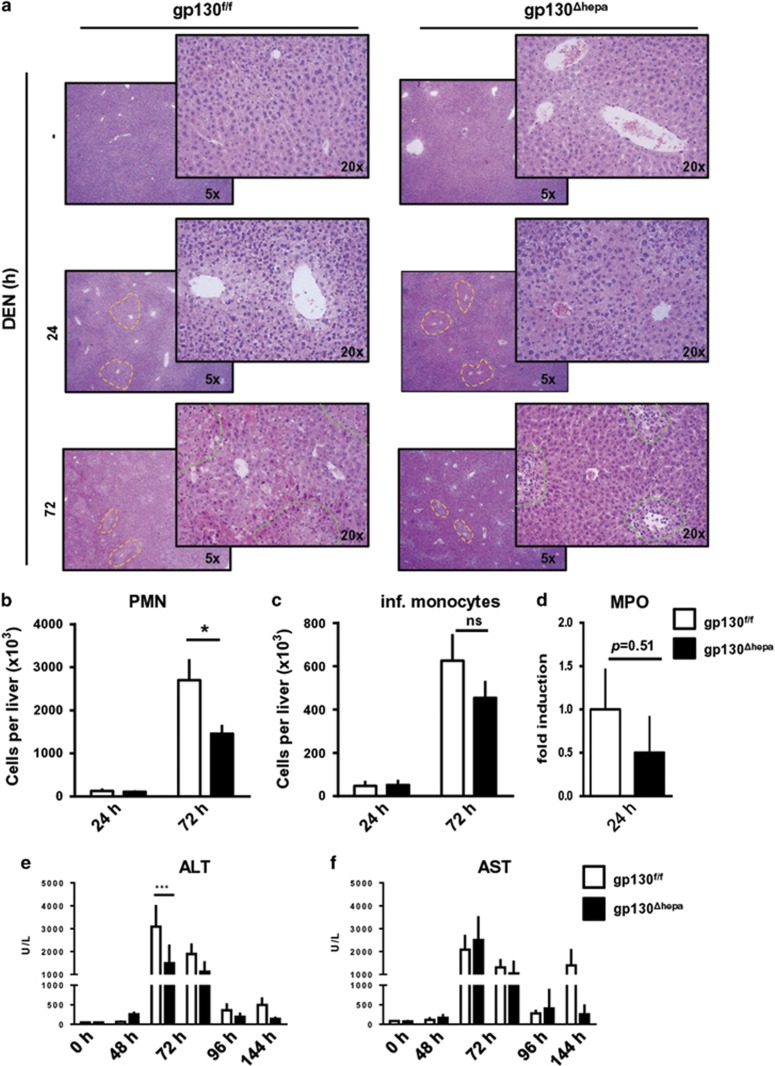
Acute DEN treatment, periportal foci and immune infiltration in hepatocyte-specific gp130 knockout mice. Gp130^f/f^ and gp130^Δhepa^ animals were treated with a single i.p. injection of DEN and killed at the indicated time-points. (**a**) Representative H&E staining of the liver sections (untreated, 24 and 72 h) (upper panel). Dotted areas in yellow represent necrotic foci. Dotted areas in green represent infiltration. Liver infiltrating PMN (**b**) and inflammatory monocytes (**c**) in 24 and 72 h DEN-treated gp130^f/f^ and gp130^Δhepa^ mice were analyzed with FACS, and quantified and represented using FlowJo. Total liver was extracted and MPO (**d**) was performed. Serum alanine transaminase (ALT) (**e**) and serum aspartate transaminase (AST) (**f**) levels were determined in gp130^Δhepa^ and gp130^f/f^ mice after DEN treatment at different time points ranging 0–144 h. All graphs show mean+SEM (*n*=5, *P<0.05; ***P<0.001).

To investigate the role of gp130 for immediate, tumor-initiating events, gp130^f/f^ and gp130^Δhepa^ mice were subjected to high-dose DEN injection (200 mg/kg i.p.) and killed up to 144 h after treatment (Figure 1a–f). Pathological examination of the H&E sections evidenced pericentral foci of small dysplastic hepatocytes and the presence of inflammatory cells observable 24 h after DEN stimulation in both groups (Figure 1a,Supplementary Figure 1). However, periportal necrosis and inflammatory cells were more pronounced in gp130^f/f^ compared with gp130^Δhepa^ livers (Figure 1a,Supplementary Figure 1). Consistently, FACS analysis showed significantly reduced infiltration of polymorphonuclear neutrophil (PMN) and a tendency for a decreased number of inflammatory monocytes in livers of gp130^Δhepa^ compared with gp130^f/f^ animals 72 h after DEN treatment (Figure 1b and c), whereas the numbers of hepatic T, B, and NK cells were unchanged (Supplementary Figure 2a and c). Moreover, 24 h after DEN treatment, myeloperoxidase activity (MPO), a marker for PMN recruitment, was slightly attenuated (*P*=0.51) in gp130^Δhepa^ compared with gp130^f/f^ animals in liver tissue homogenates (Figure 1d).

**Figure 2:**
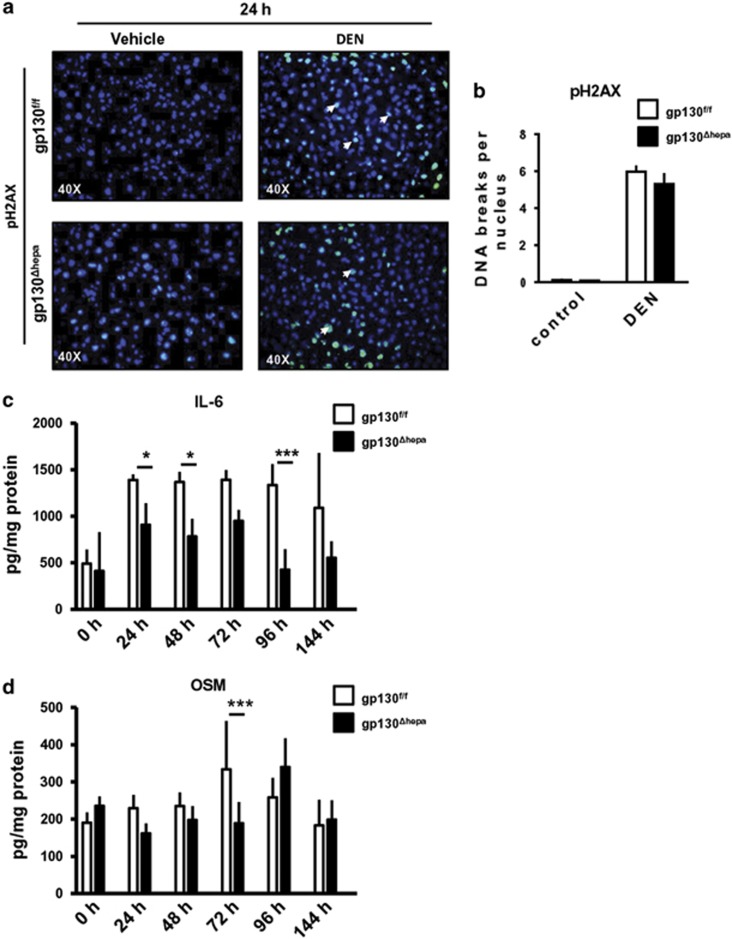
(**a**) Immunofluorescence for pH2AX on frozen liver sections 24 h after DEN treatment was performed. (**b**) Quantification of the positive cells – indicating DNA double strand breaks – was carried out. Protein lysates were extracted from livers treated with DEN for 24 h, and IL-6 (**c**) and OSM (**d**) levels were determined. Data are expressed as mean±SEM (*n*=6, *P<0.05; ***P<0.001).

Concomitant with these observations, serum alanine aminotransferase (ALT) and aspartate aminotransferase (AST) levels in gp130^Δhepa^ mice were reduced at several time points after DEN treatment (Figure 1e and f) compared with the gp130^f/f^ control group. DNA damage induced by ROS has been widely accepted as a major trigger of hepatocarcinogenesis. Phosphorylation of histone H2AX has been used as an important indicator of DNA damage.^16, 17^ Indeed, pH2AX after DNA damage has been shown to be a major determinant for cell fate.^17, 18^ Phosphorylation of H2AX (pH2AX) was markedly increased after DEN treatment. However, we could not detect a significant difference 24 h after DEN treatment between gp130^f/f^ and gp130^Δhepa^ livers (Figure 2a and b).

As we found histological differences in the inflammatory milieu of the DEN-treated livers, we examined the IL-6 protein levels in the liver (Figure 2c). IL-6 is a pleiotropic cytokine, which is involved in controlling the acute phase response during inflammation.^19, 20^ IL-6 protein levels were reduced in gp130^Δhepa^compared with gp130^f/f^livers from 24 to 144 h post treatment reaching significance at the 24, 48 and 96 h time points (Figure 2c). OSM and LIF are further members of the IL-6 cytokine family, which signal through the gp130 receptor. The protein expression of OSM was significantly higher in gp130^f/f^ compared with gp130^Δhepa^ livers, 72 h after DEN (Figure 2d) and after 40 weeks of DEN treatment (Supplementary Figure 3a). No differences were observed in LIF expression (data not shown). As gp130-driven IL-11 expression has been found to be overexpressed in inflammatory hepatocellular adenoma (IHCA),^21^ we studied whether the levels of IL-11 were affected in liver gp130-abrogated mice (Supplementary Figure 3b). Our results show a tendency toward reduced IL-11, especially 24 h and 48 h after acute DEN treatment.

### HCC initiation in gp130^Δhepa^ livers

**Figure 3:**
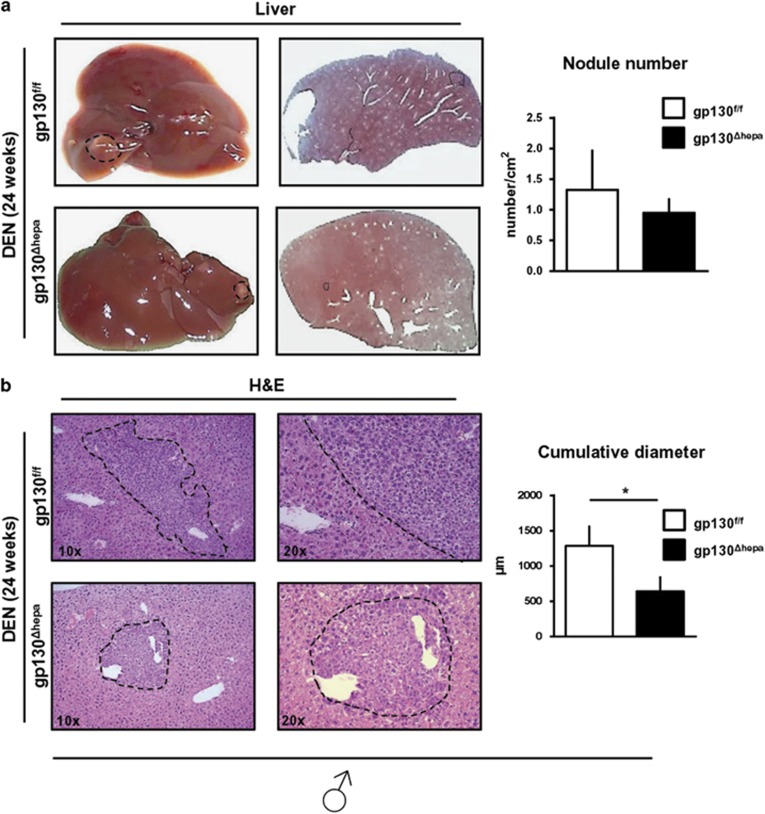
Tumor initiation in gp130^Δhepa^ livers. Male gp130^f/f^ and gp130^Δhepa^ animals were treated with a single i.p. injection of DEN and were killed after 24 weeks. (**a**) Representative macroscopic views of the livers and the number of nodules found in these livers are shown (dotted circles represent dysplastic nodules; left panel). (**b**) H&E staining was carried out on the same livers and different magnifications are shown. Nodules of random H&E slides were analyzed by an experienced pathologist in a blinded session for tumor diameter. Data are expressed as mean±SEM (*n*=5, *P<0.05).

We next investigated the impact of gp130 on tumor initiation 24 weeks after a single dose of DEN injection (25 mg/kg) in male mice. Dysplastic nodules were present in all animals killed at the 24 week time point. Lesions did not show differences in terms of appearance and localization in both groups (Figure 3a, left panel). Despite a lack of significant differences in the numbers of liver nodules, a quantitative macroscopic examination displayed a nodule diameters in gp130^Δhepa^ compared with gp130^f/f^ livers 24 weeks after DEN stimulation (Figure 3a, right panel). In addition, both the total nodule area and the number of nodules per area were smaller in gp130^Δhepa^ compared with gp130^f/f^ livers. However, these parameters did not reach statistical significance at this early time point after tumor initiation (Supplementary Figure 4a). Moreover, less visible nodules on the liver surface at the time point of explantation were found in gp130^Δhepa^ compared with gp130^f/f^ livers (Supplementary Figure 4b). Altogether, these results suggested that the loss of gp130 does not affect tumor initiation but slightly attenuated early progression of HCC after DEN-induced tumor initiation.

### Ablation of gp130 in hepatocytes reduces HCC progression in male and female mice

**Figure 4:**
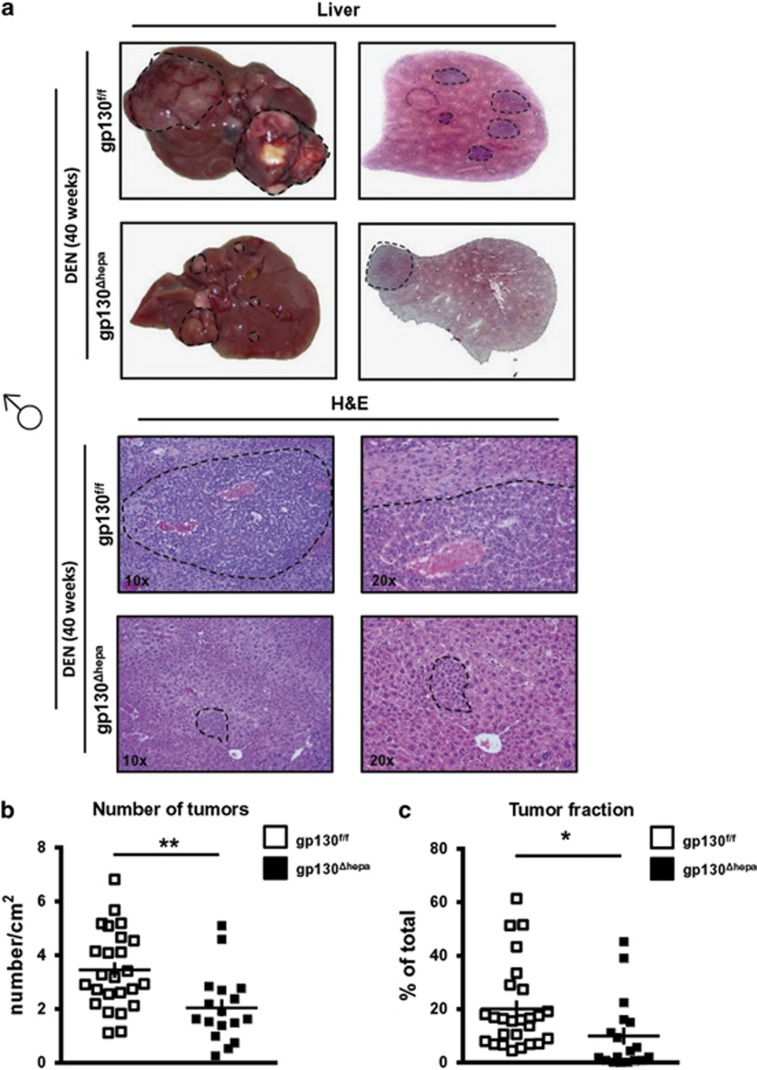
Ablation of gp130 in hepatocytes reduces HCC progression in males. Male gp130^f/f^ and gp130^Δhepa^ mice were treated with a single i.p. DEN injection and killed 40 weeks later. (**a**) Representative macroscopic views of the livers are shown (dotted circles represent dysplastic nodules; left panel). H&E staining was performed from the same livers and photographed at different magnifications (center and right panel). (**b**) Tumors of randomly chosen H&E slides were counted by an experienced pathologist in a blinded session. Total area of the liver section was measured and results calculated as tumors per cm^2^ of liver section. (**c**) The total area of the section and the area of the tumor were measured. Tumor area was calculated as the percentage of total. Data are expressed as mean±SEM (*n*=5; *P<0.05; **P<0.01).

After single low-dose DEN treatment, macroscopic HCC formation typically occurs between 35 and 52 weeks.^22^ We thus investigated tumor progression in male gp130^f/f^ and gp130^Δhepa^ livers 40 weeks after DEN injection (Figure 4). Macroscopically, tumor nodules were evident in both gp130^f/f^ and gp130^Δhepa^ livers (Figure 4a). However, the number of nodules and tumor fraction in gp130^Δhepa^ livers was significantly reduced compared with gp130^f/f^ animals at this time point (Figure 4a–c,Supplementary Figure 5a). Furthermore, these findings were associated with a reduction in tumor nodules on the surface of DEN-treated gp130^Δhepa^ livers and a clear tendency in the cumulative diameter (Supplementary Figure 5b and c), whereas the liver *versus* body weight ratio did not show statistical difference between gp130^Δhepa^ and gp130^f/f^ mice (Supplementary Figure 5d). Consistent with these data, immunofluorescence staining of frozen tissue evidenced a tendency for reduction of CD11b^+^- cell infiltration and F4/80^+^-positive staining in gp130^Δhepa^ compared with gp130^f/f^ livers as a sign of tumor-associated inflammation, 40 weeks after DEN induction (Supplementary Figure 6a and b). Altogether our findings show that gp130 in hepatocytes is involved in HCC progression.

**Figure 5:**
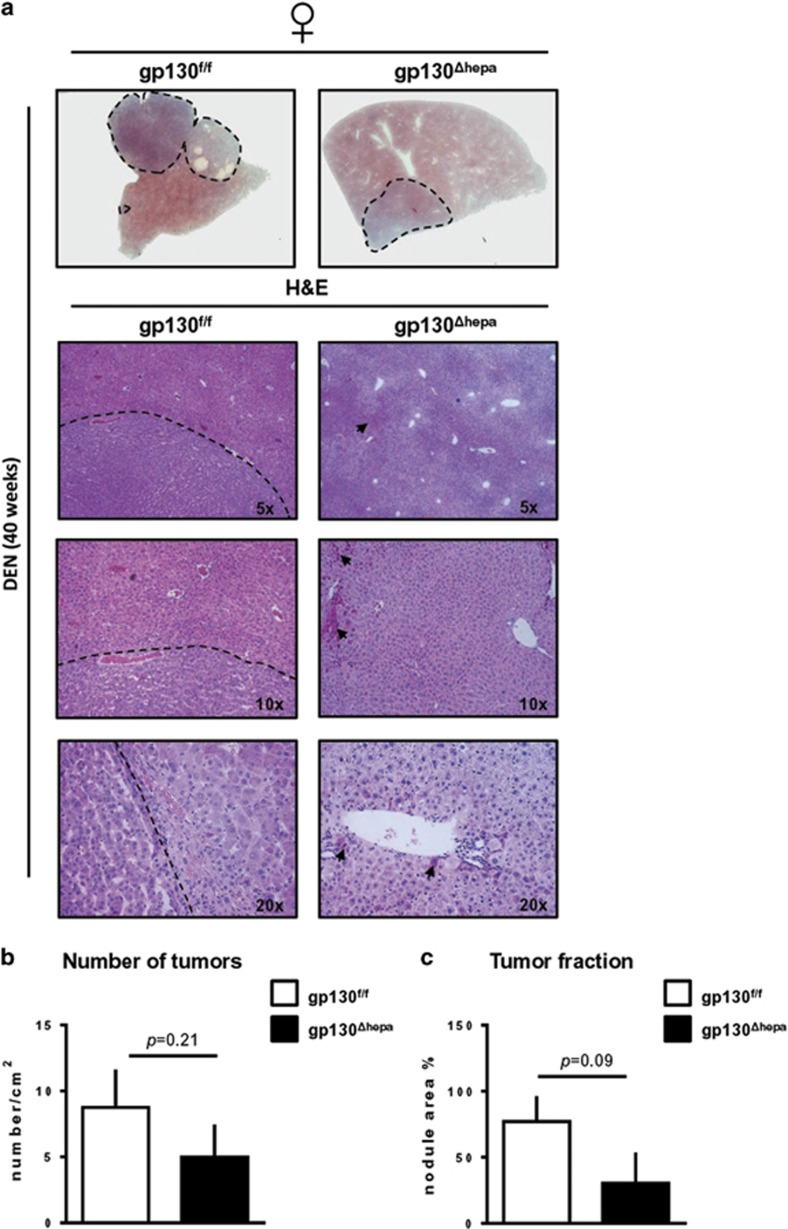
Disruption of gp130 in hepatocytes in females attenuates HCC progression. Gp130^f/f^ and gp130^Δhepa^ female mice were treated with a single i.p. DEN injection and killed 40 weeks later. (**a**) Representative macroscopic views of the livers are shown (dotted circles represent dysplastic nodules, upper panel). H&E staining was performed from the same livers and photographed at different magnifications (left and right panels). (**b, c**) The number of nodules and the tumor area were determined by an experienced pathologist in a blinded session and represented. Tumor area was calculated as the percentage of total. Data are expressed as mean±SEM (*n*=6; *P<0.05; **P<0.01).

To determine whether the previously reported gender bias in IL-6 production that accounts for the sex difference in HCC development in these animals,^14^ we examined DEN-induced carcinogenesis in female gp130^f/f^ and gp130^Δhepa^ livers, 40 weeks after DEN treatment. Macroscopical examination of liver specimens manifested smaller nodules in gp130^Δhepa^ compared with gp130^f/f^ female mice (Figure 5a, upper panel). Further examination of H&E showed presence of visible HCC areas in gp130^f/f^ female mice (Figure 5a, left panel). In contrast, gp130^Δhepa^ female livers displayed normal liver architecture with the presence of some preneoplastic cells (Figure 5a, right panel). Consistent with these findings, there was a tendency for a reduction in the number of tumors (*P*=0.21) and the tumor fraction (*P*=0.09) in gp130^Δhepa^ compared with gp130^f/f^ females, 40 weeks after DEN (Figure 5b and c). Therefore, from these results, we cannot exclude the possibility that besides IL-6 other factors may also contribute to gender disparity in liver cancer.

### ROS-dependent DNA damage is an important driver of liver injury after DEN treatment

**Figure 6:**
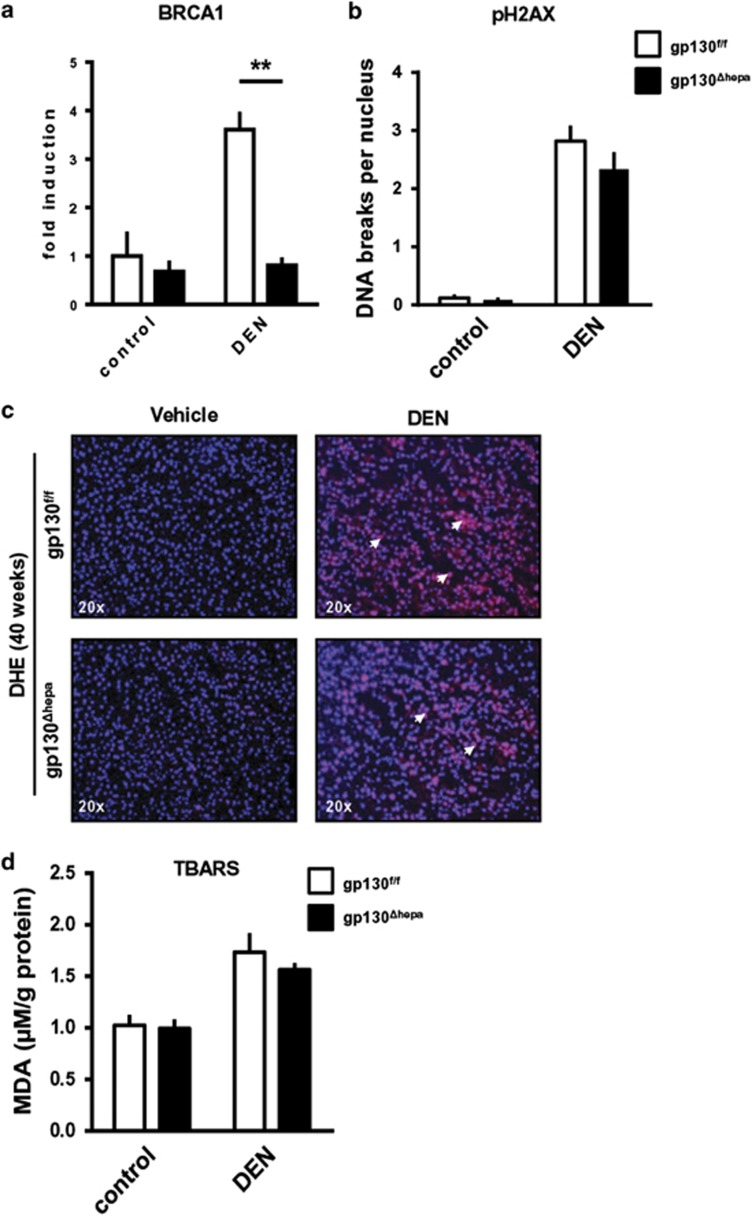
Oxidative stress plays an important role in liver injury after DEN treatment. (**a**) RNA was extracted from total liver lysates 40 weeks after DEN treatment and qRT PCR for BRCA-1 was performed. (**b**) Quantification of the pH2AX immunostaining of liver sections for pH2AX of gp130^Δhepa^ and gp130^f/f^ mice 40 weeks after DEN treatment is shown. Vehicle (saline) injected animals of both genotypes were used as controls. (**c**) Representative immunofluorescence of liver sections for dihydroethidium (DHE) of gp130^Δhepa^ and gp130^f/f^ mice 40 weeks after DEN treatment was assessed. Vehicle-injected animals of both genotypes served as controls. (**d**) Quantitative measurement of malondialdehyde (MDA) from total liver lysates of the same mice. Vehicle-injected animals of both genotypes served as controls. Data are expressed as mean+SEM (*n*=5; **P<0.01).

Maintenance of genome stability after DNA damage depends on BRCA1.^23^ We observed that 40 weeks after DEN administration BRCA1 mRNA expression was significantly elevated in DEN-induced gp130^f/f^ tumor tissue compared with gp130^Δhepa^ livers, suggesting a reduced amount of DNA double-strand breaks in gp130^Δhepa^ livers (Figure 6a). This was associated with a tendency toward reduced levels of pH2AX in gp130^Δhepa^ livers, 40 weeks after DEN treatment, although the observed differences did not reach statistical significance (Figure 6b).

Oxygen-derived species such as superoxide radicals, hydrogen peroxide, single oxygen and hydroxyl radicals have been implicated in the etiology, the initiation and the progression of HCC.^24^ Immunofluorescence staining of liver cryosections with dihydroethidium (DHE) revealed higher amounts of superoxide radicals in both gp130^Δhepa^ and gp130^f/f^ livers compared with untreated controls 40 weeks after DEN injection (Figure 6c). Furthermore, our data evidenced that liver malondialdehyde levels were not significantly changed between both groups, indicating that potential changes in overall ROS production and lipid peroxidation were not a major cause to explain differences in tumor progression (Figure 6d).

### Impaired STAT3 phosphorylation is associated with increased STAT5 activation and abrogated TGF-*β* signaling in gp130^Δhepa^ livers

**Figure 7:**
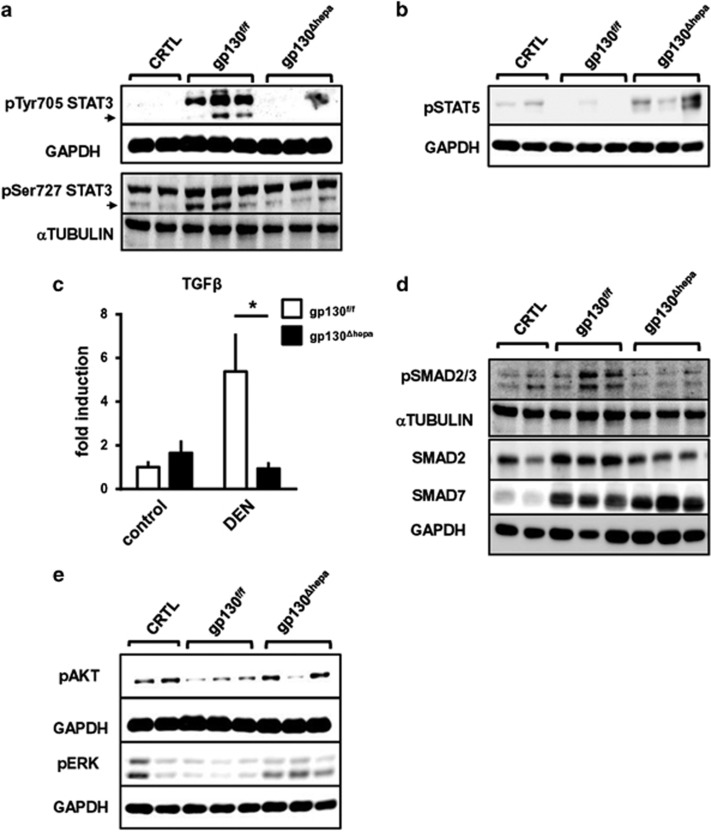
Lack of gp130 in hepatocytes is associated with STAT5 activation 40 weeks after DEN treatment. Protein levels of pTyr705 STAT3 and pSer727 STAT3 (**a**) and pSTAT5 (**b**) were determined by western blot of total liver protein lysates of 40 weeks-DEN-treated gp130^f/f^ and gp130^Δhepa^ animals. As controls, untreated animals were used. GAPDH was used as loading control. (**c**) RNA was extracted from total liver lysates after 40 weeks of DEN treatment and qRT-PCR for TGFβ was performed. (**d**) Protein levels of pSMAD2/3, SMAD2 and SMAD7 were determined by western blot in the same samples. Differences in MAPK/Ras signaling in gp130^Δhepa^ compared with gp130^f/f^ livers. (**e**) Protein levels of pAKT and pERK were determined by western blot of total liver protein lysates of 40 weeks-DEN-treated gp130^f/f^ and gp130^Δhepa^ animals. As controls, untreated animals were used. GAPDH was used as loading control. Loading control was the same for pSTAT5 and pERK as both were developed on the same membrane. Graphs show mean±SEM (*n*=3; *P<0.05).

In the mouse model, STAT3 was shown to be essential for DEN-induced carcinogenesis.^25^ Compelling molecular evidence has demonstrated a role for STAT3 in tumor initiation and progression.^19, 26, 27^ In line with these previous findings, STAT3 phosphorylation at Tyr705 and Ser727 was evident in gp130^f/f^ livers 40 weeks after DEN injection. As expected, pSTAT3 expression was strongly inhibited in gp130^Δhepa^ livers (Figure 7a, Supplementary Figure 7a and b). Noticeably, phosphorylation of serine 727 (Ser727) has been linked with neoplastic transformation of hepatocytes and also contributes to the maximal transcriptional activity of STAT3.^28^

A recent study has suggested that STAT5 might have a crucial role in hepatic tumorigenesis.^29^ However, its role in cancer development is still controversial as it might also act as a tumor inducer^30, 31, 32, 33^ as well as a tumor suppressor.^29, 34^ No STAT5 activation was detected in gp130^f/f^ livers 40 weeks after DEN treatment (Figure 7b, Supplementary Figure 7c). However, increased STAT5 phosphorylation was evident in gp130^Δhepa^ livers (Figure 7b, Supplementary Figure 7c).

Earlier results suggested a correlation between enhanced STAT3 and transforming growth factor-*β* (TGF*β*) signaling in contrast to lower STAT5 activation during growth of HCC.^35^ Thus, we examined whether TGF*β* activation was different in gp130^Δhepa^ livers. In fact, 40 weeks after DEN treatment TGF*β* expression was significantly increased in gp130^f/f^ compared with gp130^Δhepa^ livers (Figure 7c, Supplementary Figure 7d).

The SMAD protein family is frequently mutated or deleted in human cancers. In gp130^f/f^ livers, SMAD2/3 were strongly phosphorylated 40 weeks after DEN treatment, consistent with the elevated TGF*β* expression found in these livers (Figures 7c and d, Supplementary Figure 7d). Mutations in the *SMAD2* gene have a relatively high occurrence in liver cancer, and overexpression of SMAD7 has been also reported in HCC patients.^36^ Indeed, the mutant *Smads* are degraded rapidly in comparison with their wild-type counterparts.^37^ Whereas the SMAD7 expression was not changed in gp130^Δhepa^ compared with gp130^f/f^ livers, SMAD2 protein expression was downregulated in the same experimental conditions (Figure 7d,Supplementary Figures 7e and f). Thus, TGF*β*-mediated signaling pathways are attenuated in gp130^Δhepa^ livers.

### Differences in MAPK/Ras signaling in gp130^Δhepa^ compared with gp130^f/f^ livers

Finally, we investigated whether changes in STAT/TGF*β* signaling might have an impact on MAPK-dependent pathways and thus evaluated pAKT and pERK expression. Forty weeks after DEN injection pAKT and pERK expression were slightly increased in gp130^Δhepa^ compared with gp130^f/f^ livers (Figure 7e, Supplementary Figures 7g and h).

## Discussion

HCC is the most common primary malignancy in the liver and the third leading cause of cancer mortality worldwide because of the lack of effective treatment options.^38, 39, 40^ To improve the current understanding of the mechanisms of hepatic carcinogenesis, the DEN model has frequently been used to define at the molecular level HCC initiation and progression in mice,^22^ as it recapitulates the pathophysiological events of human HCC.^15^ DEN induces carcinogenesis by covalent binding and methylation of nucleic acids and proteins in hepatocytes leading to HCC development.^22^

The link between inflammation, cancer and gp130 has been well characterized in gastric and colorectal cancer.^41, 42^ Several observations have identified gp130-associated cytokines as key players in the pathophysiology of tumor initiation and progression. Among these studies, an increased IL-6 expression has been linked with HCC development.^38, 43^ Specifically, activation of IL-6 signaling through phosphorylation of STAT3 at Tyr705 and Ser727 has been detected in a wide variety of mouse and human liver cancer including IHCA.^28, 43, 44, 45, 46^ Especially in IHCA, dominant-active mutational changes in different proteins contributing to gp130-STAT3 activation are essential to lead toward benign tumor growth.^43^ However, the transition of IHCA into HCC and thus tumor initiation is not frequently found.^47^

In the present study, we observed a reduction in the DEN-induced HCC load in gp130^Δhepa^ livers. IL-6 signaling can promote tumor initiation and progression by activating multiple intracellular signaling pathways.^46^ Among those, STAT3 activation seems crucial to trigger carcinogenesis.^48^ In earlier studies, we contributed to define the role of IL-6 or STAT3 for liver injury.^11, 12^ In the present work, we addressed the relevance of the hepatocyte-specific role of gp130 for liver carcinogenesis.

Our experimental approach using conditional knockout mice presents more advantages than global mutants such as IL-6^−/−^ in which the effect of changes in tumor growth cannot be attributed to a specific cell-type. Here, activation of IL-6-dependent pathways in different cell types – especially immune cells and non-parenchymal liver cells – can also contribute to HCC development.^49^ The use of STAT3-cell-type-specific knockout mice bears limitations as gp130, the signaling molecule, is still expressed in the targeted cell. It has been shown that the intracellular gp130 docking sites are not STAT3 specific.^21^ Hence, ligand binding to the gp130 receptor also induces STAT1 activation, which has specific effects, potentially affecting the outcome of the experiments. In addition, gp130 activates other signaling cascades, and STAT3 deletion might alter receptor stoichiometry, favoring the activation of alternative pathways.

As Williams *et al.*^50^ reported that the cytotoxicity caused by injection of DEN is dose-dependent, we first performed dose–response experiments to investigate if the loss of gp130 in hepatocytes might have a role in tumor initiation and progression. We have previously shown that the lack of gp130 promotes susceptibility to LPS-induced acute liver injury.^11^ Our results indicated that the acute inflammatory response after DEN administration was ameliorated in mice with ablation of gp130 in hepatocytes. Interestingly, we found that OSM, a member of the IL-6 cytokine family, was downregulated in gp130^Δhepa^ livers, which is in agreement with previous observations that strongly link OSM to inflammation and tumorigenesis.^51^ Therefore, we asked whether these differences might be relevant to explain changes in DEN-induced tumor initiation.

We found significantly reduced dysplastic tissue area fraction and nodule size in gp130^Δhepa^ livers 24 weeks after DEN injection. However, the number of nodules in gp130^Δhepa^ livers was not significantly decreased at this time point, time point suggesting that the differences observed after acute DEN injection likely have no impact on tumor initiation.

As these results indicated that gp130^Δhepa^ had a minor effect on tumor initiation, we next investigated the role of gp130 for tumor progression (40 weeks post DEN). Here, we found that phosphorylation of STAT3 at both Tyr705 and Ser727 was abrogated in livers of gp130^Δhepa^ mice. Our findings imply that gp130 in hepatocytes is a main mediator for STAT3 activation in tumors having a significant impact on tumor progression.

Naugler *et al.*^14^ attributed the disparity in liver cancer between females and males to differences in IL-6 production by Kupffer cells in response to DEN. Accordingly, female WT mice had a much lower HCC incidence after DEN treatment compared with males, which was not the case in a IL-6^−/−^ background. It has therefore been suggested that disruption of IL-6 signaling would abolish the gender disparity in liver cancer at least in the murine DEN model.^14^

Interestingly, in our study we found that disruption of gp130 in hepatocytes also conferred some protection against DEN-induced HCC in female mice although these differences were not statistically significant owing to high variation within the animals. However, we did not observe strong differences regarding tumor incidence in our cohort of wild-type females 40 weeks after DEN. Altogether, from our own data we cannot exclude the possibility that besides IL-6, other factors may also contribute to gender disparity in liver cancer. This conclusion is in agreement with previous work published by the group of Klaus Kaestner^52^ and others,^36, 47, 53^ diminishing the enthusiasm about a therapeutic use of estrogens in the clinic.

The changes in tumor progression were associated with a significant reduction in the expression of several gp130 target genes. Interestingly, we could not detect any differences in oxidative stress or immune cell infiltration between gp130^Δhepa^ and gp130^f/f^ mice, factors that are known to be associated with cancer progression.^54^ Therefore, we searched for an alternative mechanism that could mediate this effect.

DNA damage has an important role in tumor growth of DEN-induced carcinogenesis. DNA-repair mechanisms, such as non-homologous end joining, recombination repair and base excision repair, counter-act the mutagenic effect of DEN.^55^ However, no significant differences in DNA damage were found after acute DEN-induced injury using different doses. In fact, after 24 weeks of treatment with DEN, we only observed a tendency toward reduced DNA damage. Nevertheless, DNA repair depends on BRCA1,^23^ which was significantly diminished in gp130^Δhepa^ mice. Thus, these results suggest that the degree of DNA damage directly after DEN injection is not significantly altered by the lack of gp130 expression.

In search of potential mechanisms explaining reduced HCC progression in gp130^Δhepa^ mice, we found increased STAT5 phosphorylation in gp130^Δhepa^ liver. As STAT5 activation has been shown to trigger tumor growth in a TGF*β*-dependent manner,^29^ we examined the TGF*β* pathway in more detail. We found significantly decreased TGF*β* expression in gp130^Δhepa^ livers associated with reduced protein levels of SMAD2 and pSMAD2/3. TGF*β*1 has been detected in blood and urine of patients with HCC, and its presence is associated with poor prognosis.^56^ Our data therefore suggest that attenuated TGF*β* signaling contributes to reduced HCC progression in gp130^Δhepa^ livers after DEN injection. However, even though the interplay between STAT3 and STAT5 has been described in the literature,^57, 58^ it remains unclear how loss of STAT3 activity leads to STAT5 activation.

IL-6/STAT3 activity often correlates with tumorigenesis and poor prognosis in humans, processes which are linked to the gp130-signaling pathway.^3^ Moreover, somatic mutations coding for constitutively activated gp130 have been detected in hepatocellular adenoma.^43^ Indeed, clinical trials with IL-6 antibodies have shown good antitumor efficacy leading, yet, massive systemic elevations in IL-6. To overcome this problem, drugs which prevent binding of IL-6 to IL-6 R, or IL-6 neutralizing antibodies are currently under investigation. However, the major hurdle here is to overcome the global IL-6 blockade caused by these drugs. To achieve this goal, a possible therapeutic strategy solution has been recently published by Bartneck *et al.,*^49^ with the use of gold nanorods, which represent a relatively novel class of nanoparticles that hold significant potential for delivering drugs such as gp130-signaling pathway blockers to specific cell types (e.g., hepatocytes).

**Figure 8:**
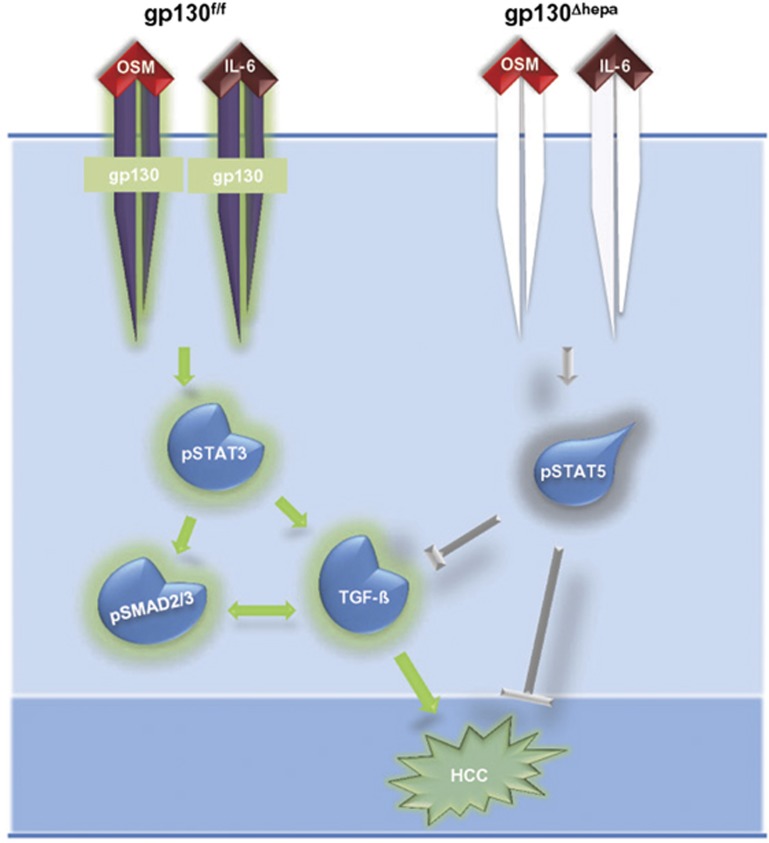
Gp130 in hepatocytes contributes to tumor progression in the DEN model. In the presence of gp130 in hepatocytes, DEN induces IL-6 and OSM signaling through gp130 activating STAT3, TGFb-dependent pathways and HCC progression. The absence of gp130 in hepatocytes results in decreased IL-6 and OSM signaling and constitutive phosphorylation of STAT5 occurs, which impairs TGFb-dependent mechanisms, thus attenuating HCC development.

We conclude that gp130 in hepatocytes contributes to tumor progression but not to tumor initiation in the DEN model. In the presence of gp130 in hepatocytes, DEN induces IL-6 and OSM signaling through gp130-activating STAT3, TGF*β-*dependent pathways and HCC progression. Conversely, the absence of gp130 in hepatocytes results in decreased IL-6 and OSM expression, constitutive phosphorylation of STAT5 and subsequently impaired TGF*β*-dependent signaling, thereby attenuating HCC development (Figure 8). Thus, gp130 in hepatocytes seems an interesting therapeutic target for blocking progression of HCC.

## Materials and methods

### Animals and induction of tumorigenesis

For our study, we used gp130^f/f^ mice in a C57BL/6 background. In these mice, exon 16 encoding the gp130 transmembrane domains is flanked by loxP sites as described previously.^59^ Gp130^f/f^ mice were crossed with transgenic mice expressing Cre recombinases under control of the albumin promoter/enhancer and the *α*-fetoprotein enhancer as previously described,^12^ resulting in gp130^Δhepa^ mice with specific deletion of gp130 in liver parenchymal cells (hepatocytes and cholangiocytes). Mice were housed in 12-h light/dark cycles, with free access to food and water and were treated in accordance with the criteria of the German administrative panel on laboratory animal care and approved by the local Animal Care Committee. At least five animals per time point were analyzed. All experiments were repeated at least three times. Male and female mice were injected intraperitoneally with 200 mg/kg body weight (time points 0–144 h) or 25 mg/kg body weight (time points 24–40 weeks) of DEN (Sigma-Aldrich, Munich, Germany) at 14 days of age. Mice were killed at 0 h, 24 h, 48 h, 72 h, 96 h or 24 and 42 weeks after injection, respectively. Vehicle-injected (saline) 44-week-old male mice served as controls. All animals were injected within a time period of 3 months with the same DEN lot, ensuring best comparability between treated groups.

### Analysis of liver tumors

Each liver lobe was separately photographed with the Leica Z16 APO macroscope from every side. Tumor formations >1 mm in diameter were counted and measured using the calibrated Diskus software (Hilgers, Königswinter, Germany). Conventional Haematoxyline & Eosin (H&E) stainings were evaluated for signs of malignancies, and malignant areas were measured using the Diskus software in a blinded session.

### Gene expression analysis

Liver tissue was shock-frozen in liquid nitrogen and stored at −80 °C. RNA was purified by pegGOLD (peqLab, Erlangen, Germany) using standard protocols, and complementary DNA was generated from 1 mg RNA using a complementary DNA synthesis kit (Roche, Basel, Switzerland). Quantitative real-time (RT-PCR was performed using SYBR green reagent (Invitrogen, Darmstadt, Germany) reactions were carried out twice in quintuples, and GAPDH values were used to normalize gene expression. Primer sequences are available on request.

### Immunoblot analysis

Liver tissues were homogenized in ice-cold NP40-Buffer containing 50 mM Tri-HCl (pH 7.5), 150 mM NaCl, 0.5% NP40 and 50 mM NaF freshly supplemented with Complete Mini (Roche), PhosSTOP (Roche), 1 mM orthovanadate and 1 mM pefablock. Protein concentrations were determined by BIO-RAD protein assay (BIO-RAD Laboratories GmbH, Munich, Germany). Samples were separated by SDS-PAGE and transferred to a cellulose membrane and probed with antibodies for pAKT, pERK, pTyr705 STAT3, pSer727 STAT3, pSTAT5, pJNK and pSMAD2/3 (Cell Signaling, Danvers, MA, USA). As secondary antibodies, anti-rabbit-HRP (Cell Signaling) and antimouse-HRP (Santa Cruz, Heidelberg, Germany) were used. GAPDH from AbD SeroTec (Düsseldorf, Germany) was used as loading control. Analysis of intrahepatic IL-6 (Abcam, Cambridge, UK) and OSM (R&D Systems, Minneapolis, MN) was performed in duplicates (*n*=6) using a murine ELISA kit.

### Immunofluorescence

For immunofluorescence staining, cryosections were fixed in 4% PFA for 10 min, rinsed in phosphate-buffered saline (PBS)-Tween for 10 min and blocked with 10% goat serum for 1 h. Incubation with primary antibodies CD11b (BD Pharmingen, Heidelberg, Germany), F4/80 (MCA497, Serotec, Raleigh, NC, USA), pH2AX (Cell Signaling) and DHE (Invitrogen, Grand Island, NY, USA) was performed at 4 °C overnight. Slides were rinsed in PBS and incubated with appropriate fluorescence-labeled secondary antibodies (AlexaFluor 488 and 564, Invitrogen) for 1 h at room temperature. Slides were washed twice in TBS-Tween and mounted with Vectashield containing DAPI (Linaris, Wertheim, Germany). All sections were analyzed and documented using an Imager Z1 fluorescence microscope together with Axiovision software (Carl Zeiss, Jena, Germany).

### Flow cytometry analysis

Immune cells from whole liver were isolated as described recently^54^ and stained with fluorochrome-conjugated antibodies (CD45, CD11b, CD11c, Ly6G, Gr1, CD3, CD19, NK1.1, all BD Biosciences, Heidelberg, Germany) and HOECHST for live–dead differentiation. All samples were acquired by flow cytometry (FACS Canto II; BD Biosciences) and analyzed using the FlowJo software (Tree Star Inc, Ashland, OR, USA). Gating strategies are shown in Supplementary Figure 3A and B.

### Serum parameters

ALT and AST were measured in the Central Laboratory Facility at University Hospital RWTH Aachen according to standard procedures.

### Statistical analysis

All experimental data are expressed as mean and error bars are shown as S.E.M. Differences between more than two groups were assessed by analysis of variance, and between two groups by two-tailed unpaired Student's *t*-test (GraphPad), except where indicated otherwise.

## Abbreviations

HCC: hepatocellular carcinoma
IL-6: interleukin-6
Gp130: glycoprotein 130
Gp130^Δhepa^: hepatocyte-specific gp130 knockout mice
Gp130^f/f^: Control floxed animals
DEN: diethylnitrosamine
H&E: haematoxyline & eosin (H&E)
PMN: polymorphonuclear neutrophil
IL-6R*α*: interleukin-6 receptor alpha
OSM: oncostatin M
LIF: leukemia inhibitory factor
IL-11: interleukin-11
ROS: reactive oxygen species
DNA: deoxyribonucleic acid
H2AX: histone 2AX
MPO: myeloperoxidase activity
PMN: polymorphonuclear neutrophil
BRCA1: breast cancer 1, early onset
DHE: dihydroethidium
MDA: malondialdehyde
STAT: signal transducer and activator of transcription
TGF*β*: transforming growth factor-*β*
JNK: c-Jun N-terminal kinase
ERK: extracellular signal-regulated kinase-1
G6P: glucose-6-phosphatase
IHCA: inflammatory hepatocellular adenoma
LPS: lipopolysaccharide.
