# Supplementary material for: Lack of gp130 expression in hepatocytes attenuates tumor progression in the DEN model
Source: Cell Death Dis. 2015 Mar 5;6(3):e1667–. doi: 10.1038/cddis.2014.590 (PMC4385909; doi:10.1038/cddis.2014.590)

Lack of gp130 expression in hepatocytes attenuates tumor progression in the DEN model

Maximilian Hatting, Michael Spannbauer, Jin Peng, Malika Al Masaoudi, Gernot Sellge, Yulia A. Nevzorova, Nikolaus Gassler, Christian Liedtke, Francisco Javier Cubero, Christian Trautwein

SUPPLEMENTARY FIGURE LEGENDS

Supplementary Figure 1 $Gp130^{f/f}$ and $gp130^{\Delta hepa}$ animals were treated with a single *i.p.* injection of DEN and sacrificed at the indicated time-points. (a) Representative H&E staining of the liver sections (untreated, 48 h, 96 h and 144 h). Dotted areas in yellow represent necrotic foci. Dotted areas in green represent infiltration.

Supplementary Figure 2 (a) FACS gating strategy for PMNs and inflammatory monocytes. (b) FACS gating strategy for T-, B-, and NK-cells. (c) Liver infiltrating T-, B-, and NK-cells in 24 h and 72 h DEN-treated challenge $gp130^{f/f}$ and $gp130^{\Delta hepa}$ mice. Data are expressed as mean + SEM (n=4).

Supplementary Figure 3 (a) RNA was extracted from $gp130^{f/f}$ and $gp130^{\Delta hepa}$ total liver lysates, 40 weeks after DEN treatment and qRT-PCR for OSM was performed. (b) cDNA was obtained from 24 weeks samples of $gp130^{f/f}$ and $gp130^{\Delta hepa}$ and the

mRNA expression of IL-11 was evaluated. Data are expressed as mean + SEM (n=5).

Supplementary Figure 4 (a) Liver section area and tumor area were measured, and tumor area was calculated relative to total area (%). (b) Livers were explanted and nodules on the surface were counted at the same time point. All graphs show mean + SEM (n=3).

Supplementary Figure 5 (a) Mean diameter of tumors as measured on randomly chosen H&E slides of each animal in a blinded session. (b) Cumulative diameter of all tumors evident on randomly chosen H&E slides of each animal in a blinded session. (c) Livers were explanted and nodules on the surface were counted by an experienced pathologist. (d) The percentage between the liver and the body weight ratio was quantified and represented. All graphs show mean + SEM (at least 10 mice were used).

Supplementary Figure 6 (a) Representative immunofluorescence of liver sections for CD11b of gp130$^{\Delta hepa}$ and gp130$^{f/f}$ mice 40 weeks after DEN treatment was performed. Quantification of CD11b immunofluorescence displayed as positive cells per view-field. (b) Representative immunofluorescence of liver sections for F4/80 of gp130$^{\Delta hepa}$ and gp130$^{f/f}$ mice 40 weeks after DEN treatment was performed. Quantification of F4/80 immunofluorescence using Image J© and represented as % positive area per view-field. For both immunostainings, vehicle injected animals of both genotypes served as controls. All pictures show 20X magnification. All graphs show mean + SEM (n=5, *p<0.05).

Supplementary Figure 7 The expression of pTyr705 STAT3 (a), pSer727 STAT3 (b), pSTAT5 (c), pSMAD2/3 (d), SMAD (e), SMAD7 (f), pAKT (g) and pERK (h) were determined by Western blot analysis and quantified by Image J software related to their respective house-keeping genes (GAPDH or αTubulin), and the untreated controls (* $p<0.05$, ** $p<0.01$).

Supplementary Figure 1

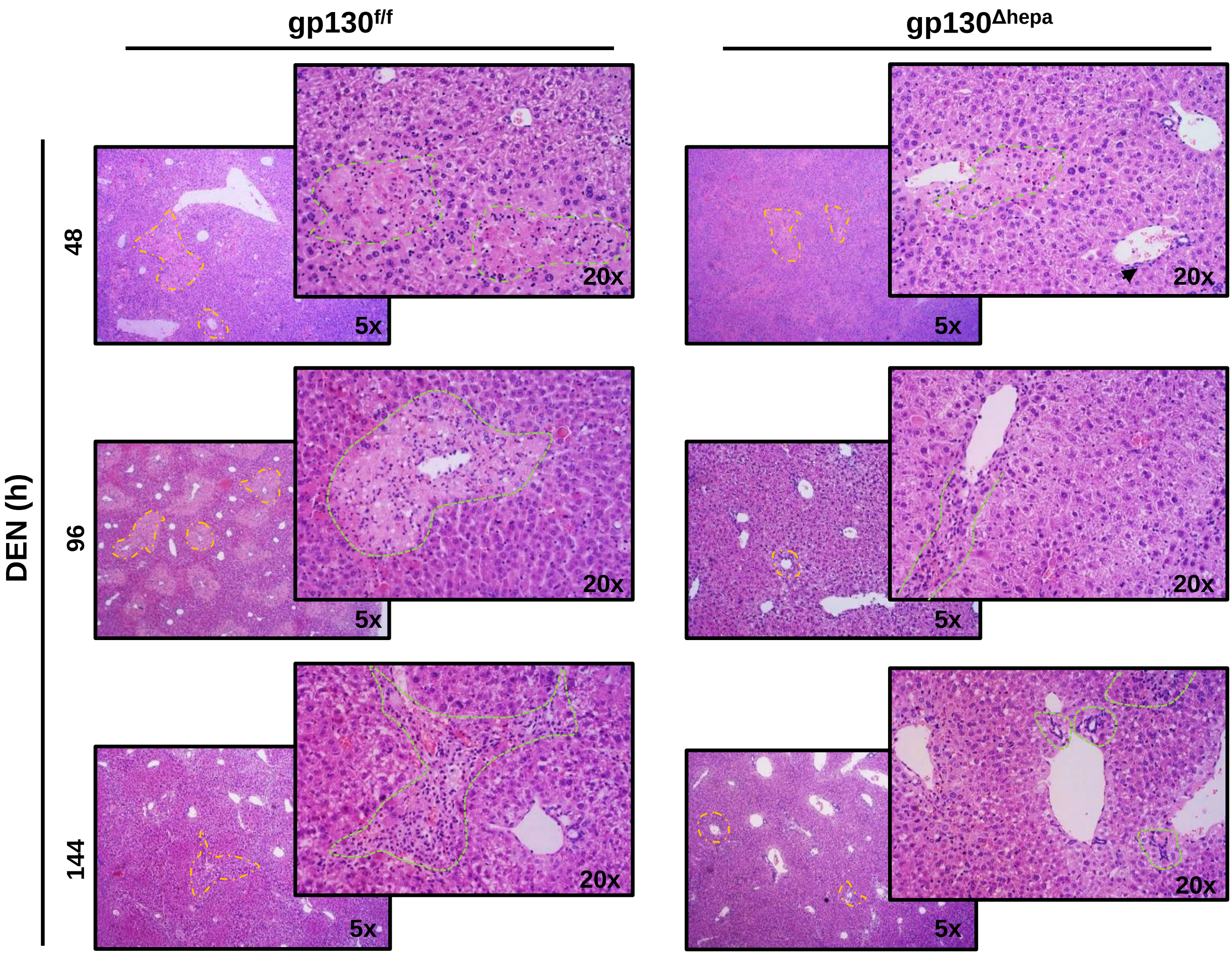

Supplementary Figure 2

a

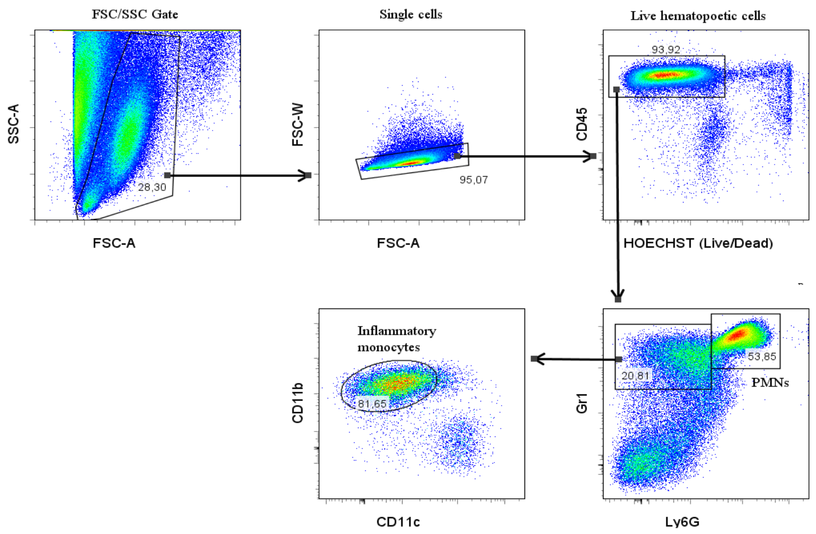

b

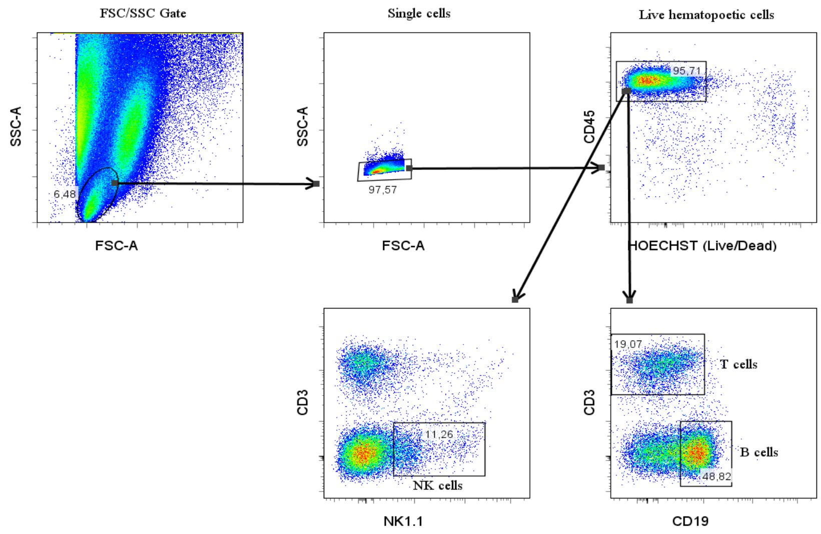

c

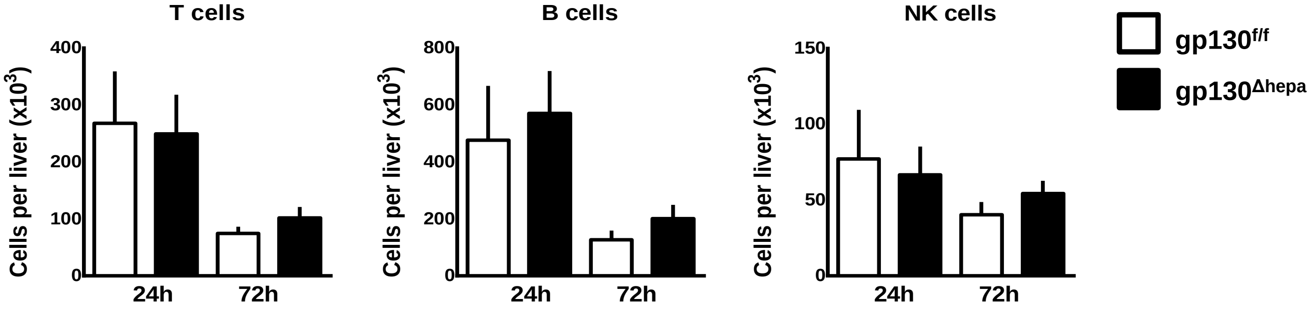

Supplementary Figure 3

a

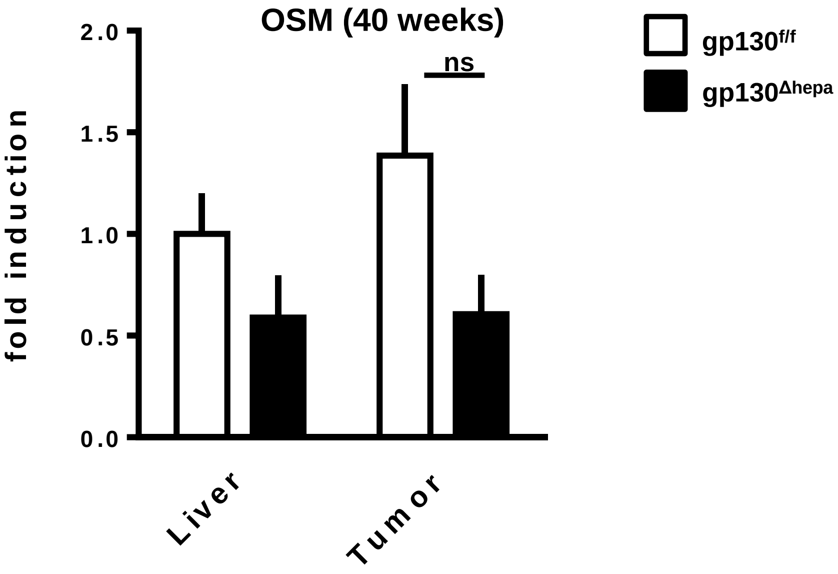

b

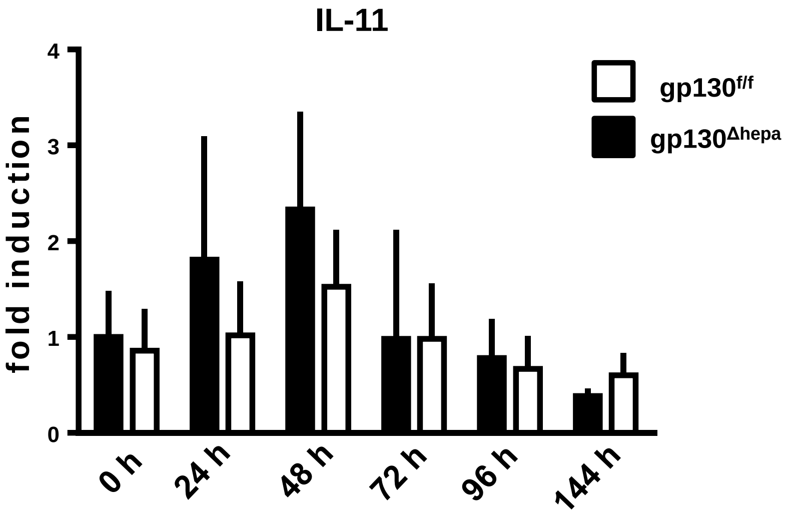

Supplementary Figure 4

a

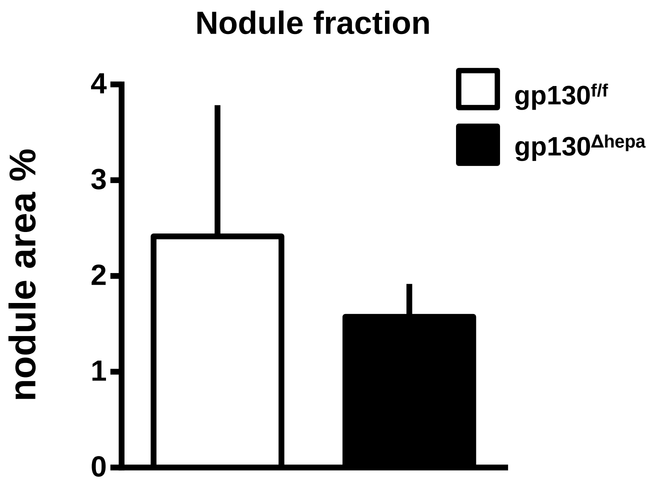

b

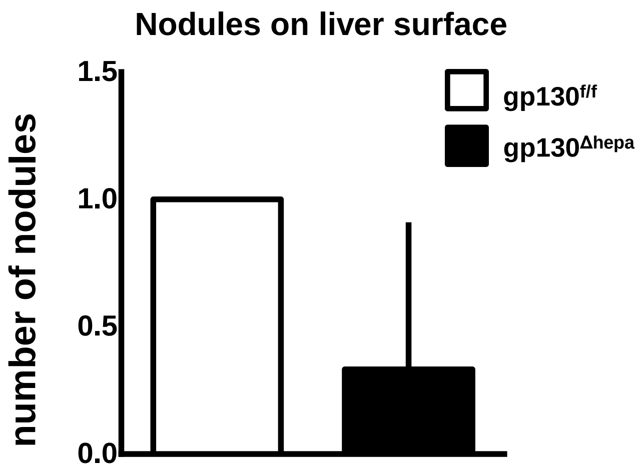

Supplementary Figure 5

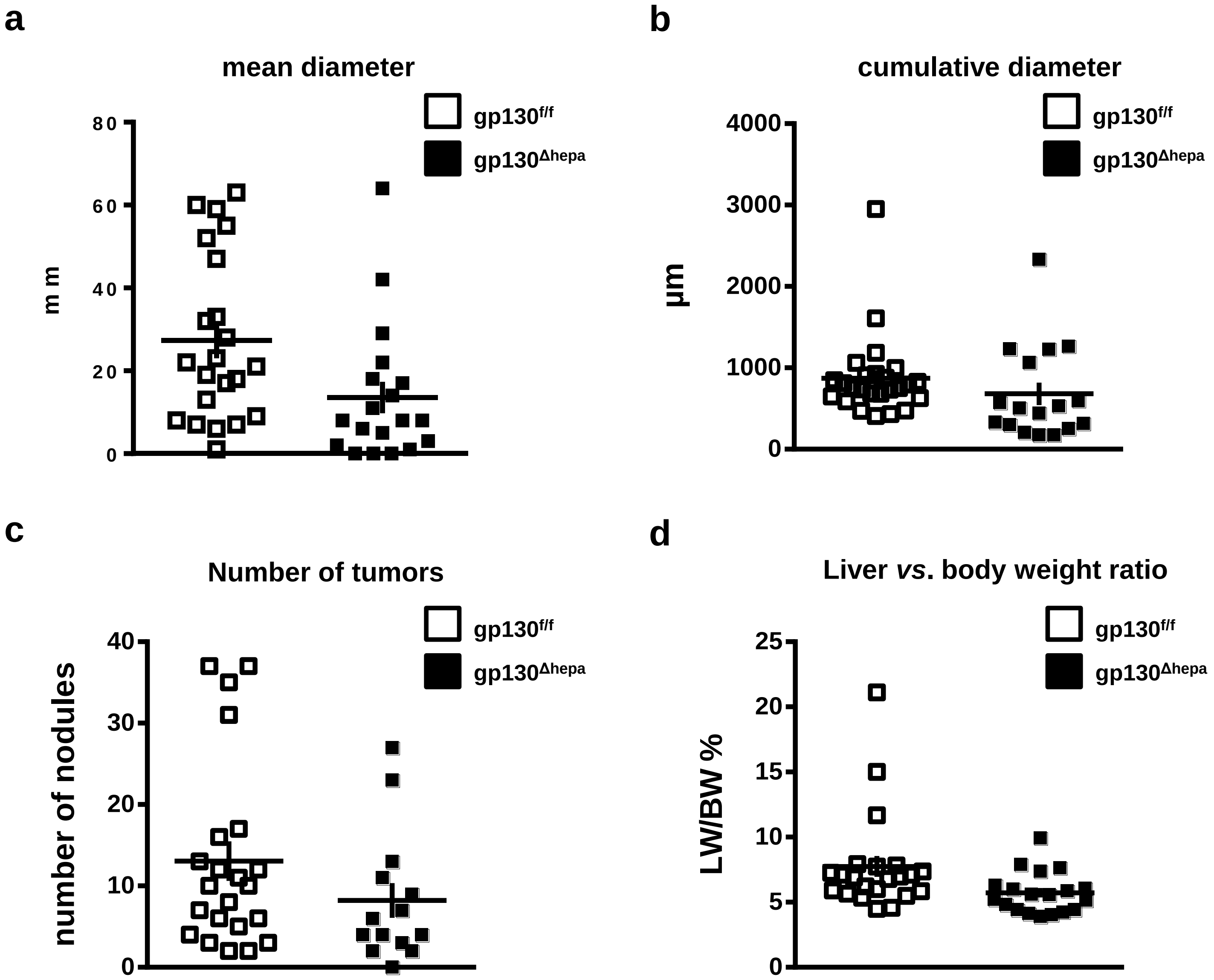

Supplementary Figure 6

a

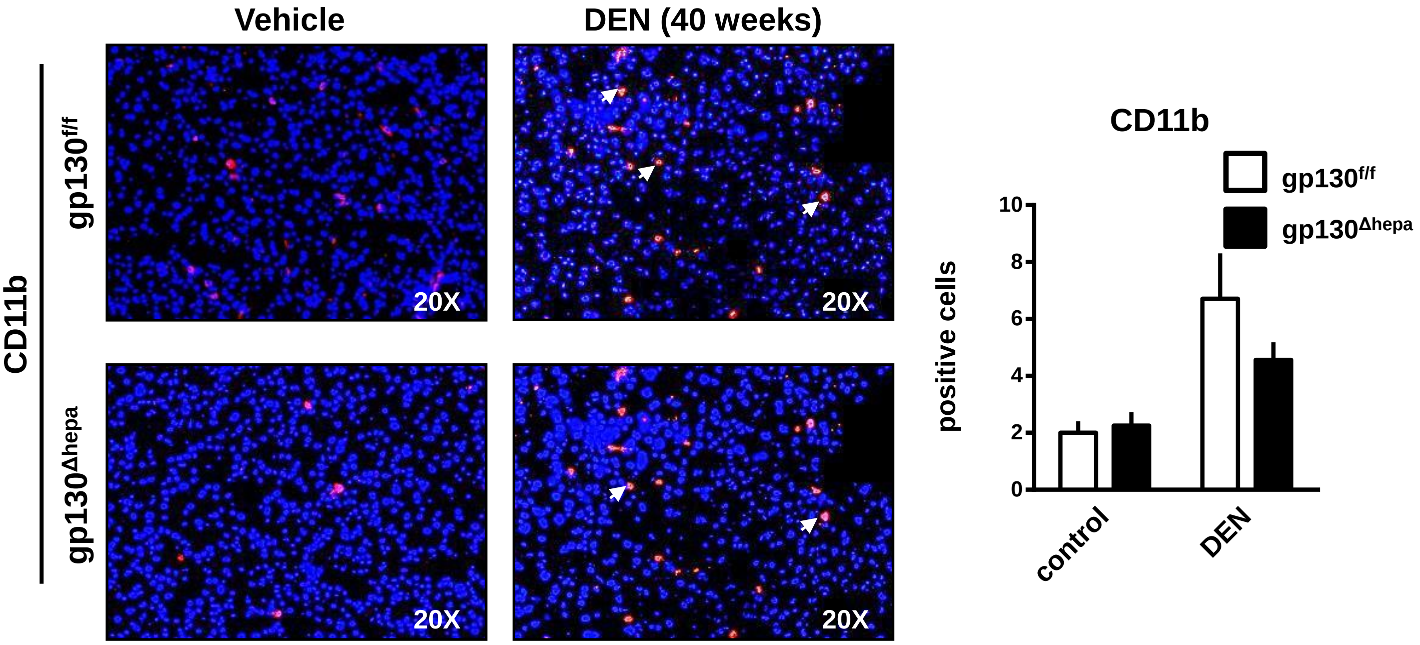

b

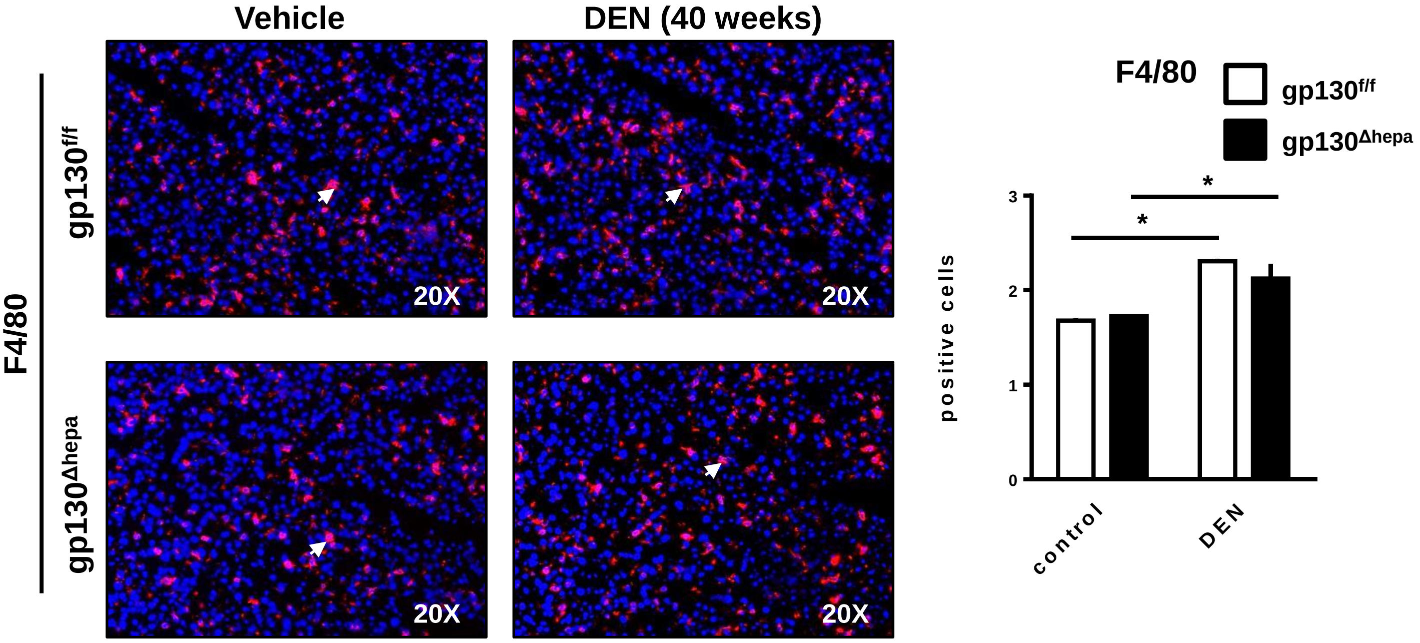

Supplementary Figure 7

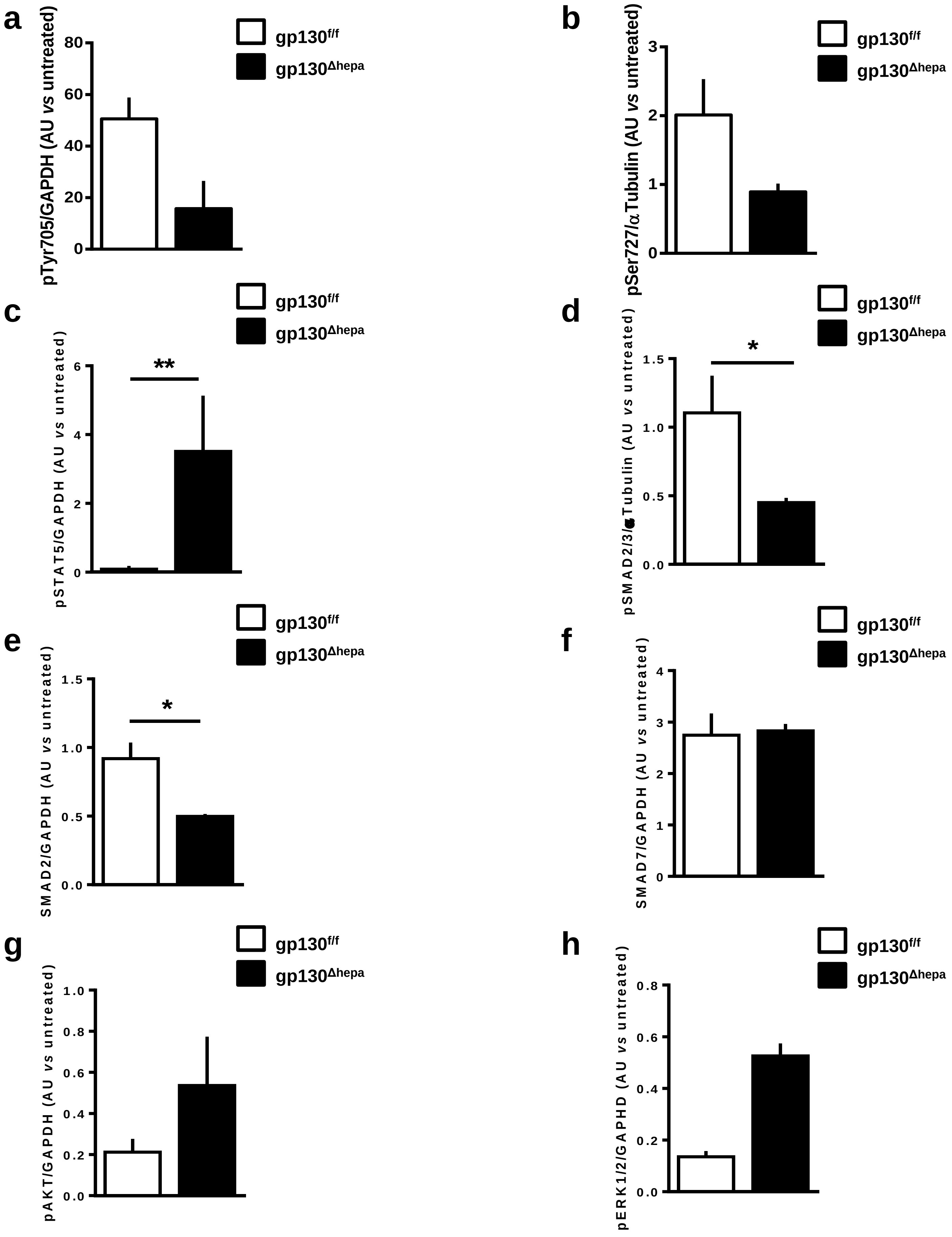

Supplement: Supplementary Information [file cddis2014590x1.pdf]
